# Biomolecular Condensates in Myeloid Leukemia: What Do They Tell Us?

**DOI:** 10.1097/HS9.0000000000000923

**Published:** 2023-06-27

**Authors:** Zivojin Jevtic, Melanie Allram, Florian Grebien, Juerg Schwaller

**Affiliations:** 1 Department of Biomedicine (DBM), University Children’s Hospital Basel, University of Basel, Switzerland; 2 Institute for Medical Biochemistry, University of Veterinary Medicine, Vienna, Austria; 3 St. Anna Children’s Cancer Research Institute (CCRI), Vienna, Austria

## Abstract

Recent studies have suggested that several oncogenic and tumor-suppressive proteins carry out their functions in the context of specific membrane-less cellular compartments. As these compartments, generally referred to as onco-condensates, are specific to tumor cells and are tightly linked to disease development, the mechanisms of their formation and maintenance have been intensively studied. Here we review the proposed leukemogenic and tumor-suppressive activities of nuclear biomolecular condensates in acute myeloid leukemia (AML). We focus on condensates formed by oncogenic fusion proteins including nucleoporin 98 (NUP98), mixed-lineage leukemia 1 (MLL1, also known as KMT2A), mutated nucleophosmin (NPM1c) and others. We also discuss how altered condensate formation contributes to malignant transformation of hematopoietic cells, as described for promyelocytic leukemia protein (PML) in PML::RARA-driven acute promyelocytic leukemia (APL) and other myeloid malignancies. Finally, we discuss potential strategies for interfering with the molecular mechanisms related to AML-associated biomolecular condensates, as well as current limitations of the field.

## INTRODUCTION

An increasing number of deep sequencing studies unraveling the genomic landscape of acute myeloid leukemia (AML) indicated that the conversion from a normal to a transformed state of hematopoietic stem and progenitor cells is the consequence of a relatively small number of genetic alterations that can be divided in to at least 11 functional classes.^1^ Notably, many of them rewire gene expression by producing mutated variants of master transcriptional regulators of myelopoiesis, transcriptional coregulators, chromatin-modifiers, regulators of RNA-splicing, or fusion proteins.^1,2^ Biochemical characterization of AML-associated oncoproteins revealed that they often act in large and most likely dynamically-formed multiprotein complexes that either directly or indirectly interact with chromatin. Several of these complexes alter gene expression programs by modifying chromatin through posttranslational modification (PTM) of histones and/or DNA methylation, resulting in increased or reduced access of transcription factors (TFs) to their cognate target sequences.^3,4^ The concept that mutated TFs and coregulators drive malignant transformation is not limited to hematological malignancies, as similar mutational scenarios were identified, for example, in mesenchymal tumors driven by EWS::FLI or TLS/FUS::ERG fusion oncogenes.^5–7^

In contrast to multiprotein complexes, which are dispersed within the intracellular space, certain proteins, RNAs, and DNA are found highly enriched within one or several intracellular locations. These micron-scale membrane-less compartments, often termed as biomolecular condensates, carry out essential biological functions such as ribosome biogenesis or transcription.^8,9^ Recent reports suggested that the function of transcriptionally active AML-driving proteins is carried out within biomolecular condensates, and their formation was proposed to be pivotal for the induction and maintenance of the disease.^8–10^ While several of these oncoproteins are the result of well-established AML-associated gene fusions, condensate formation has been also linked to the function of normal proteins, whose expression level, but not structure, is affected by AML-driving mutations^11,12^ (Suppl. Table S1). Biomolecular condensates are generally formed through weak, multivalent interactions between intrinsically disordered regions (IDRs) of proteins, thus causing their local, subcellular enrichment.^10^ Importantly, nucleic acids influence the condensation behavior of proteins carrying IDRs and structured domains (eg, reader modules).^13,14^ Together, the capacity of biomolecules to interact at the right place and time, and at right concentrations, leads to the creation of functional, microscopically visible puncta-like compartments that are composed of proteins, RNA, and DNA.

First molecular insights into how biomolecular condensation can mediate malignant transformation were obtained from studies of the EWS::FLI1 fusion protein, which is a molecular hallmark of Ewing’s sarcoma.^5^ EWS::FLI1 acts as an oncogenic TF that forms chromatin-associated nuclear condensates at highly repetitive GGAA microsatellite regions.^15^ Activation of the oncogenic transcriptional program of EWS::FLI1 depends on the recruitment of the chromatin remodeling BRG/BRM-associated (BAF) protein complex. This interaction is mediated by phosphorylation of tyrosine residues in the IDR of the EWS moiety, necessary for the multimerization of EWS::FLI1 fusion with wild-type EWSR1 and for the formation of nuclear condensates that are composed of EWS::FLI1 and its interactors.^5^ Apart from oncogenes, also tumor-suppressive activity has been associated with biomolecular condensates. The UTX (ubiquitously transcribed X chromosome tetratricopeptide repeat protein) H3K27 demethylase acts as a tumor suppressor by regulating genome-wide histone modifications and higher-order chromatin interactions in a condensation-dependent manner.^15^ Strikingly, UTX mutations, which are found in several cancers, occur most frequently in the IDR of the protein, resulting in disrupted nuclear condensation of UTX and impaired tumor suppressive function.

## FORMATION AND FUNCTION OF BIOMOLECULAR CONDENSATES

**Figure 1.**
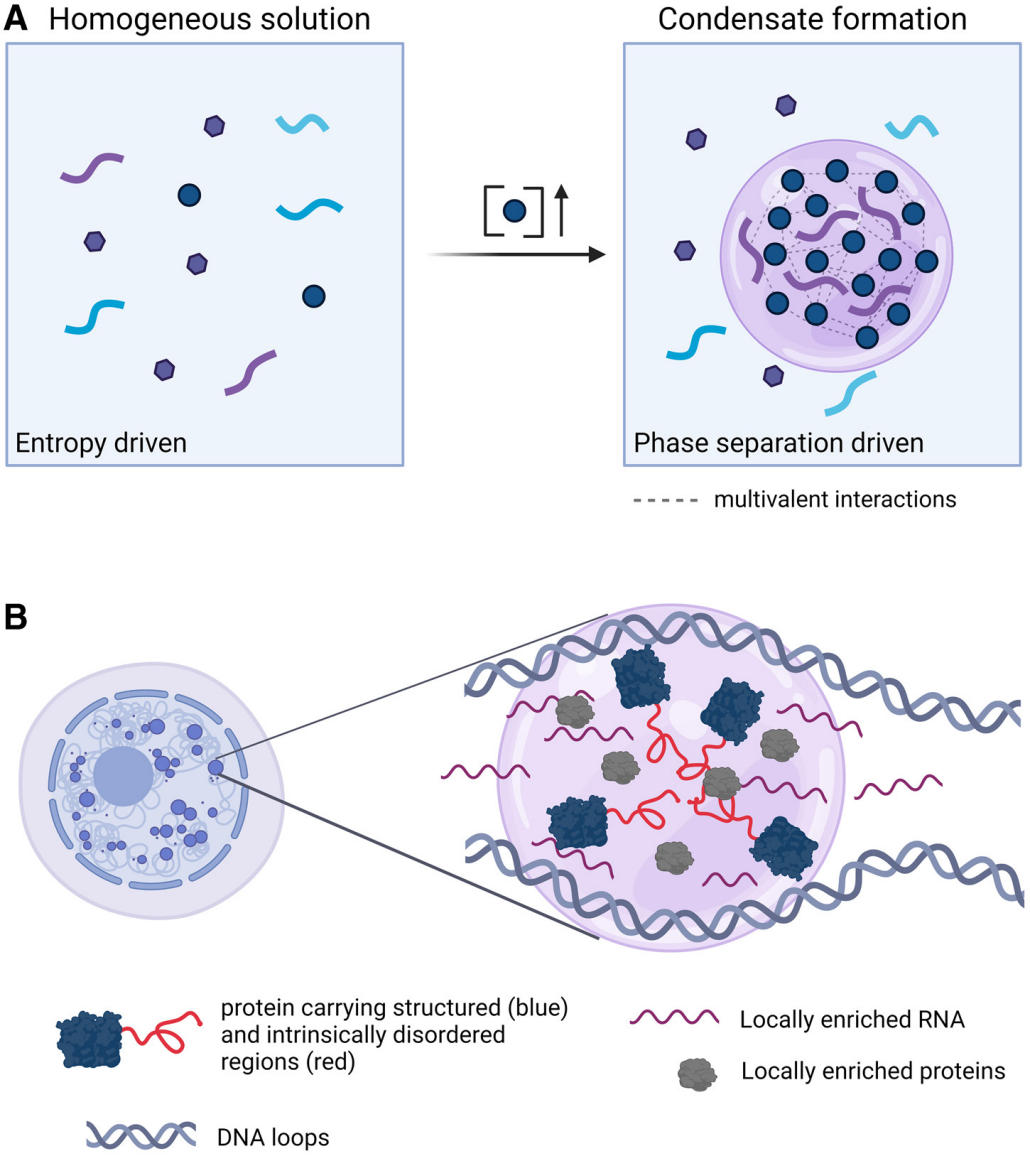
**Schematic representation and functions of phase separation in vitro and in vivo.** (A) In an entropy-driven homogeneous solution, different molecular species remain dispersed. If one (or more) of the species reaches a critically high concentration, it becomes more thermodynamically favorable for these molecules to segregate into a phase-separated condensate. (B) Within cells, multivalent associations between proteins carrying IDRs induce high local concentration of factors that compose membrane-less subcellular compartments. In the nucleus, proteins, RNAs, and DNA interact to form these functional subcompartments. IDRs = intrinsically disordered regions.

Efficient cellular logistics is based on the organization of biochemical processes within specific compartments that receive, modulate, and respond to molecular signals to maintain essential processes. While many subcellular compartments (organelles) are enclosed by lipid membranes (eg, in mitochondria or the Golgi apparatus), membrane-less compartments such as the nucleolus can also achieve local concentration of factors to mediate specific functions.^16,17^ How do membrane-less compartments maintain their structure and function within cells remains one of the fundamental questions in cell biology with direct implications for diseases including cancer. A phase separation-based hypothesis has become, for many, the default explanation for the mechanism by which membrane-less compartments are formed to enable spatiotemporal regulation of molecular processes.^18,19^ Phase separation is a physical process in which 2 (or more) molecular species segregate into distinct phases instead of remaining mixed with other molecules in the initially entropy-driven homogeneous solution.^20^ De-mixing occurs when one of the molecular species reaches saturating concentration (also referred as supersaturation) at which it becomes thermodynamically favorable for those molecules to segregate into a separated phase-condensate^21^ (Figure 1A). Condensation is driven by weak and multivalent interactions among saturated molecular species, while the surface tension transforms a condensed phase into a spherical shape droplet.^19^ Biophysical laws and principles of phase separation in cells have been widely reviewed, and will not be further discussed here.^20,22^ Importantly, alternative mechanisms, which do not involve phase separation have also emerged as explanations for the functional organization of factors in the absence of membranes. For instance, compartmentalized replication centers that are established upon infection with herpes simplex virus, however, display characteristics that are distinct from phase-separated biomolecular condensates.^23^

Within cells, weak multivalent associations between proteins and/or nucleic acids induce high local concentrations of factors, which become visible by confocal or high-resolution microscopy as membrane-less subcellular compartments.^24,25^ In vitro studies suggest that multivalent homotypic and heterotypic interactions mediated by protein IDRs drive their potential to establish biomolecular condensates.^26,27^ Importantly, IDRs are enriched for the residues targeted by PTMs, which were also shown to regulate condensate formation or disassembly.^28–30^ A well-known example are the N-terminal tails of histones, which are disordered in isolated histone proteins^31^ and in the crystal structure of the nucleosome core particle.^32^ In vitro reconstituted polynucleosome chains undergo histone tail-driven condensation, which can be modulated by acetylation or binding of multibromodomain proteins.^33^ Moreover, formation of RNA condensates in the absence/presence of proteins has been described.^34,35^ In vitro reconstitution of RNA/protein condensates showed that RNA concentration has a potent effect on their formation and physicochemical properties.^36,37^ Analogous to proteins, RNA modifications were shown to induce condensate formation and maintenance.^38^ Apart from the PTMs and RNA modifications, formation and dynamics of cellular condensates is regulated by other ATP-driven processes involving chaperones^39^ and helicases.^40^ Intracellular biomolecular condensates were proposed to contain 2 major components, including scaffolds, which are essential for the formation and maintenance of condensates, and clients, which, although dispensable for condensate formation, interact with scaffolds and can be sequestered or released from the compartment.^8,10^ All classes of biomolecules including RNA, DNA, and proteins may function as scaffolds and/or clients, and both scaffolds and clients can be important for the function of condensates^14^ (Figure 1B).

Membrane-less nuclear organelles such as nucleoli, Cajal bodies, nuclear speckles, paraspeckles, promyelocytic leukaemia protein (PML) nuclear bodies, nuclear stress bodies, as well as cytoplasmic processing bodies (P bodies) and stress granules, have been characterized with regard to subcellular localization and function, enrichment in proteins carrying IDRs, and high concentration of specific classes of RNA and/or DNA.^41^ A growing body of evidence suggests that common physicochemical features among the previously characterized and more recently identified membrane-less compartments represent the basis for shared mechanisms of their formation through biomolecular condensation.^41^ For instance, it has been demonstrated in vitro and supported by in vivo experiments that the nucleolus, a nuclear compartment that produces ribosomal RNAs and mediates ribosome assembly, represents a biomolecular condensate that is composed of IDR-containing proteins including nucleophosmin (NPM1) and fibrillarin (FBL), RNA (rRNA and small nucleolar RNAs), and DNA.^42–44^ Similarly, biomolecular condensation of RNA polymerase II, TFs, transcriptional co-activators, and enhancer RNAs was proposed to mechanistically underlie the clustering of DNA enhancer elements into super-enhancers, which govern the transcription of genes required for the establishment and maintenance of cellular identity.^45^

In summary, compartmentalization of proteins, RNA, and DNA through biomolecular condensation has emerged as a pivotal mechanism for the organization and regulation of cellular processes (Figure 1). The general role of biomolecular condensation in the context of malignancy has been discussed in several recent reviews.^8–10^ Here, we highlight the current knowledge about the function and localization of oncogenic proteins in the context of biomolecular condensates in AML.

## CONDENSATE FORMATION BY AML-ASSOCIATED FUSION ONCOPROTEINS

In over one third of de novo AML cases, the cancer cells carry structural chromosomal aberrations. If these alterations involve an in-fame fusion of protein-coding regions, oncogenic fusion proteins with strong transforming activity can be expressed.^46,47^ Intriguingly, fusion proteins tend to be structurally disordered at their breakpoint regions and contain significantly more IDRs than the rest of the human proteome.^48^ It has been suggested that through its partially disordered structure, the fusion of 2 unrelated protein parts appears like a natural protein, thus bypassing surveillance and degradation by the proteasome.^48^ Specific combinations of structural domains produce AML-associated fusion proteins, which are almost universally composed of IDRs and/or oligomerization domains fused to chromatin- or RNA-binding domains.^49^ The interplay between the 2 fused segments establishes a neomorphic function that can underlie the transforming activity of oncogenic fusion proteins.^50,51^

**Figure 2.**
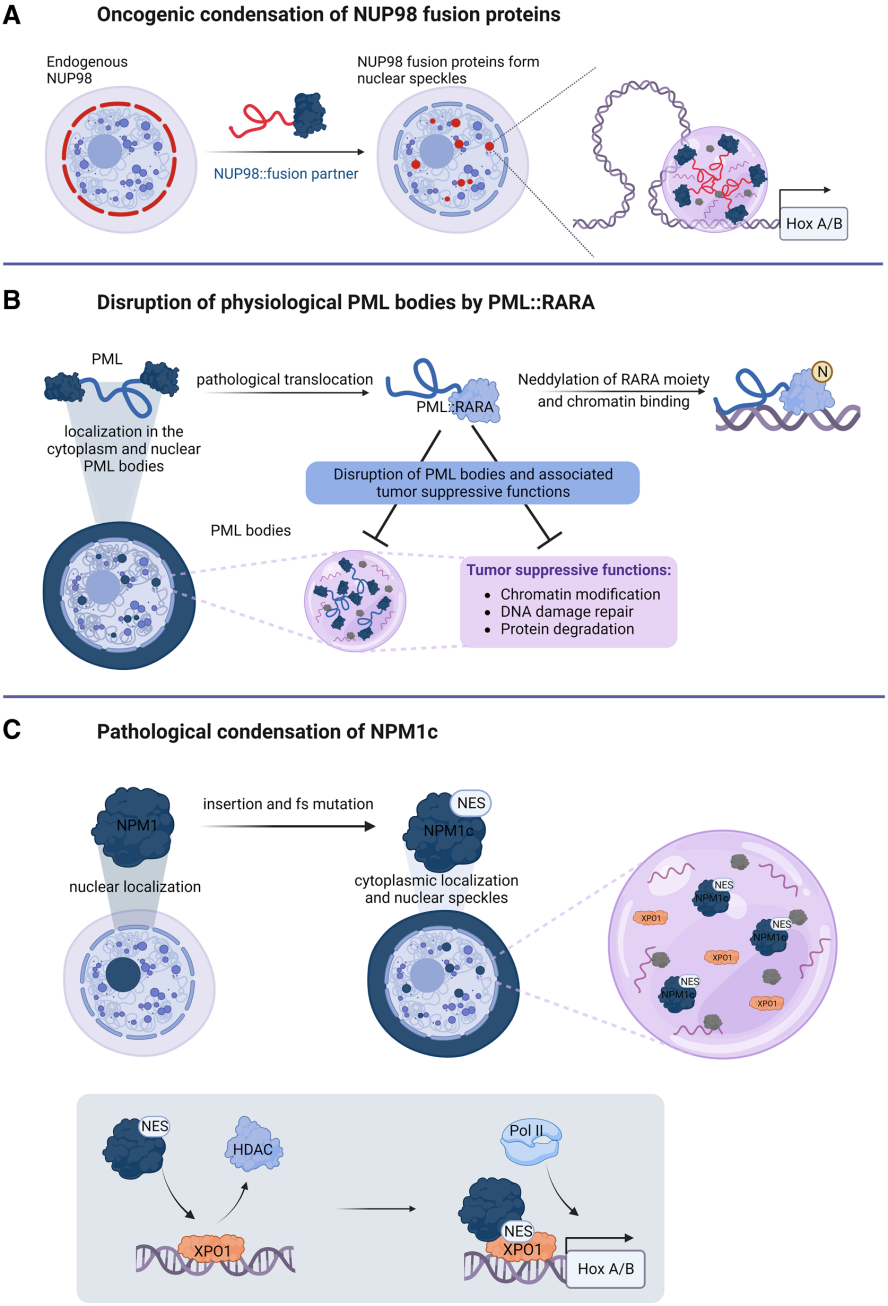
**Pathological condensation of AML oncoproteins.** (A) Endogenous NUP98 localizes to the nuclear membrane whereas oncogenic NUP98 fusion proteins form nuclear condensates that are required for the induction of leukemic transcriptional programs and transformation. (B) Endogenous PML forms nuclear bodies through oligomerization. Nuclear PML bodies have tumor-suppressive functions by regulating chromatin modifications, DNA damage repair, and protein degradation. In APL, the PML::RARA fusion protein disrupts the formation of physiological PML bodies and impedes their tumor-suppressive functions. PML::RARA is neddylated at the RARA moiety leading to enhanced chromatin binding and inhibition of PML:PML interactions. (C) NPM1 is mainly localized to the nucleolus, where it is involved in ribosome biogenesis. Insertion and frameshift mutations (fs) lead to insertion of a NES, causing the relocation of NPM1c to the cytoplasm and to nuclear speckles. Within nuclear condensates NPM1c binds to chromatin that is prebound by XPO1, removes repressive HDACs, and further recruits RNA-Pol II to induce leukemic gene expression. AML = acute myeloid leukemia; HDAC = histone deacetylase; NES = nuclear export signal; PML = promyelocytic leukemia protein.

Fusion proteins involving nucleoporin 98 (NUP98) are recurrently found in a variety of hematological malignancies and are molecular hallmarks of pediatric AML.^52,53^ In all >30 NUP98 fusion variants that have been detected until now, the N-terminal part of NUP98 is universally included. This part of the protein contains IDRs that consist of a series of repeats of FG/GLFG amino acids that are separated by an RNA-binding GLEBS domain.^54^ In the context of wild-type NUP98, the IDRs engage in multivalent, hydrophobic interactions, which lead to multimerization and phase separation of NUP98 and other nucleoporins in the nuclear pore complex. This creates the barrier function of the nuclear pore that is essential for regulated protein translocation between the nucleus and the cytoplasm.^55^ Exogenous overexpression of NUP98 caused the formation of intranuclear bodies that were termed GLFG-bodies, as the N-terminal IDR of NUP98 was required for their formation.^56^ NUP98 fusion partners can be divided into proteins bearing homeobox (HOX) domains that bind DNA and non-HOX partners that often contain chromatin-binding and/or chromatin-modifying domains (eg, plant homeodomains, acetyltransferase, or methyltransferase domains).^54^ Recent studies have shown that oncogenic NUP98 fusion proteins localize to nuclear biomolecular condensates in cell line models as well as in patient-derived AML cells^57,58^ (Figure 2A). The FG repeat-containing IDR within the NUP98 N-terminus was both essential for establishing NUP98 fusion condensates and for leukemic transformation.^58–60^ Characterization of the protein interactomes of 5 different NUP98 fusions by affinity purification and mass spectrometry identified a shared set of 157 interactors that was enriched for proteins that have previously been shown to form biomolecular condensates (eg, FUS, HNRNPA1, and GAR1).^60^ Functional annotation of core NUP98-fusion protein interactors revealed an enrichment of proteins involved in transcriptional regulation and RNA metabolism. Earlier studies suggested that NUP98 fusions induce transcriptional activation of *HOXA* and *HOXB* locus genes, which are by default repressed during normal hematopoietic lineage commitment and thus block differentiation and mediate aberrant stemness, which contributes to oncogenic transformation.^61^ NUP98::HOXA9-containing biomolecular condensates promoted long-distance looping between enhancers and promoters of oncogenes that drive leukemogenesis, such as *PBX3* and *HOX* genes (Figure 2A). These studies also showed that fusing an unrelated IDR of the FUS gene or an artificial peptide containing 39 FG repeats to known NUP98 fusion partners like KDM5A and HOXA9 was sufficient to activate a leukemia-associated transcriptional program and induce leukemia in vivo. These experiments provided proof of concept that the combination of IDRs with chromatin-regulating domains results in neomorphic proteins that drive oncogenic transformation through condensate formation.^50,58,60^ Importantly, while focusing on the NUP98::NSD1 fusion, we observed that the composition, but not the formation of condensates, was essential to maintain the transformed phenotype, thus pointing toward specific functions of proteins within fusion protein-containing condensates.^62^

Characteristic combinations of structural domains that include IDRs fused to chromatin-binding domains are also observed in the family of MLL-fusion proteins, which are found in 5%–10% of acute leukemias.^63^ MLL1 (also known as KMT2A) is a large (3969 amino acids) histone-lysine-N-methyltransferase acting as a transcriptional regulator. Despite the fact that a fragment of about 120 kDa of the MLL N-terminus is preserved in all MLL fusion proteins, and only 2 of its domains, which mediate chromatin binding, are essential for the transforming activity of MLL-fusion proteins.^64^ The AT-hook sequence recruits the LEDGF protein (lens epithelium derived growth factor), which contains a PWWP histone reader domain recognizing the H3K36 di/trimethylation mark that is found at actively transcribed chromatin.^65^ LEDGF together with Menin (MEN1) forms an MLL1-LEDGF-MEN1 ternary complex, which tethers MLL fusions to chromatin.^66,67^ In addition, the CxxC zinc finger motif in MLL1 binds to unmethylated CpG islands within transcriptionally active promoters.^68^ Indeed, it is possible to create functional MLL-fusion proteins by joining isolated PWWP (interacting with AT-hook sequence) and CxxC domains to a fusion partner.^69^ Although over 100 fusion partners have been identified, MLL is fused to AF4, AF9, AF10, or ENL in >80% of cases.^70^ Importantly, all these fusion proteins interact with the same transcriptional complexes and bear highly homologous IDRs such as ANC1 homology domains in AF9 and ENL, binding to AF4.^64^ ENL has been shown to compartmentalize with AF4 within condensates established by the super elongation complex, which is essential for the release of paused RNA polymerase II (Pol II) and transcriptional elongation.^71^ The capacity of MLL-fusion partners to cross-interact within transcription elongation complexes results in transcriptional amplification and elevated expression of preactivated genes.^64^ Earlier studies documented subnuclear compartmentalization of MLL fusion proteins,^72^ while colocalization of AF4 and AF9 in AF4 bodies was discovered a decade later.^73^ However, it remains unclear whether these structures represent biomolecular condensates, and it is also not known whether they are important for the oncogenic function of MLL fusion proteins. Thus, while in the context of NUP98 fusion proteins, the role of the conserved N-terminus is to induce the formation of biomolecular condensates, the critical function of the conserved MLL N-terminus is to elicit chromatin targeting of MLL-fusion proteins, and biomolecular condensation might be induced by C-terminal fusion partners of MLL. Importantly, however, NUP98 fusion proteins were also reported to interact with MLL complex members, and MLL was proposed to be essential for the development and maintenance of NUP98-rearranged AML.^74,75^

In contrast to the NUP98 and MLL oncofusions, the PML::RARA fusion protein disrupts the formation of physiological biomolecular condensates. PML::RARA is found in the vast majority of patients with acute promyelocytic leukemia (APL).^76,77^ In the fusion protein, the N-terminus of the promyelocytic leukemia (PML) protein is fused to the C-terminus of retinoic acid receptor alpha (RARA), resulting in the formation of an aberrant transcriptional regulator. Wild-type PML localizes within PML bodies in the nucleus (Figure 2B). PML bodies have functions in chromatin remodeling, DNA repair, and protein degradation, and have been associated with tumor suppression.^78^ These biomolecular condensates are established by PML oligomerization via interactions of RBCC domains (RING finger domain followed by 2 cysteine-histidine-rich B-box domains [B] and an alpha-helical coiled-coil domain), followed by SUMOylation of PML and its interacting proteins.^79^ In presence of the PML::RARA fusion protein, PML oligomerization is hindered due to aberrant neddylation of the RARA moiety, which leads to enhanced DNA-binding of the fusion protein.^80^ Therefore, the expression of PML::RARA disrupts the formation of physiological PML bodies, thus interfering with their tumor-suppressive functions and causing aberrant transcriptional programs^81^ (Figure 2B). Overall, these findings suggest that restoring biomolecular condensation of PML bodies through oligomerization of wild-type PML might eliminate the pathogenic effect of the PML::RARA fusion.

## CONDENSATE FORMATION BY OTHER AML-ASSOCIATED ONCOPROTEINS

Several recent studies demonstrated that high expression of proteins with IDRs other than fusion proteins play an important role in AML (Suppl. Table S1). In about 30% of all AML patients, the mutational insertion of 4 base pairs in the last exon of the nucleophosmin (*NPM1*) gene results in an in-frame shift generating a nuclear export signal in the NPM1 protein. While the wild-type NPM1 protein is a nuclear protein with several proposed functions, including ribosome biogenesis and maintenance of genomic stability,^82^ the mutated form (termed NPM1c) relocates to the cytoplasm^82,83^ (Figure 2C). Despite the abundance of the NPM1c mutation in AML, the molecular mechanism underlying its oncogenic activity is incompletely understood. It was proposed that the mislocalization of NPM1c causes cytoplasmic sequestration of important myeloid TFs, thereby preventing them from being active in the nucleus.^84,85^ However, NPM1c-mutant AML is characterized by high expression of *HOXA* and *HOXB* genes,^86^ and it was unclear if the mutant NPM1c protein plays a direct role in this process. Despite being mainly cytoplasmic, a fraction of NPM1c is detected in the nucleus, where it forms microscopically visible phase-separated condensates through IDR-mediated homo and heterotypic interactions^87,88^ (Figure 2C). Two recent studies demonstrated that NPM1c chromatin binding recruits high local concentrations of RNA-Pol II and members of transcription elongation complexes into biomolecular condensates at transcriptionally active chromatin loci, such as the *HOXA/B* gene cluster.^89,90^ NPM1c binds to genomic sites with existing active transcription that are preoccupied by exportin 1 (XPO1) in AML blasts, but not in healthy CD34^+^ hematopoietic stem and progenitor cells. Chromatin binding of NPM1c disrupts the repressive activity of local histone deacetylase (HDAC) complexes that is required for physiological differentiation of myeloid blasts, but conversely establishes super-enhancer-like condensate structures that maintain high *HOXA/B* transcription^89^ (Figure 2C). The authors proposed an insightful model in which preleukemic mutations affecting *DNMT3A*, *TET2*, and *IDH1/2* prepare the chromatin landscape for the step-wise binding of XPO1, which is followed by mutation of NPM1c that often occurs as a late event.^89^

Another AML-associated and biomolecular condensate-forming oncoprotein is meningioma-1 (MN1), a transcriptional coactivator that is frequently overexpressed in AML and also involved in rare AML-associated chromosomal translocations.^91,92^ Intriguingly, most of these translocations do not result in the expression of a fusion but cause aberrantly high expression of the MN1 protein.^93,94^ For instance, the t(12;22)(p13;q11) translocation involving *MN1* and *ETV6* genes results in hijacking of enhancer regions within and downstream of the *ETV6* locus that drive high expression of full-length MN1.^11^ Interestingly, the MN1 protein bears one of the longest polyQ-stretches found in the human proteome.^95^ PolyQ-stretches are IDRs that can mediate the formation of higher-order complexes or self-aggregation in various cellular processes.^96^ The MN1 polyQ-stretch is essential for redistribution of overexpressed MN1 into pathologic nuclear condensates.^11^ Within these nuclear condensates, MN1 localizes to active enhancer regions, where it recruits the SMARCA4 subunit of the BAF chromatin remodeling complex through its polyQ-stretch IDR. In turn, the polyQ-stretch was essential for leukemogenesis driven by MN1 overexpression through the activation of enhancers that regulate a leukemogenic transcriptional program.^11^

Apart from aberrant regulation of transcriptional processes, leukemogenesis can also be driven by posttranscriptional mechanisms such as dysfunctional mRNA modifications. A number of studies discovered a critical role for m^6^A-methylation in leukemogenesis.^97^ Cheng and coworkers recently reported aberrantly high expression of *YTHDC1* (YTH domain containing 1) in cells from AML patients.^12^ YTHDC1 is a reader protein that recognizes m^6^A-methylation in RNA that is mediated by the METTL3 (methyltransferase 3), METTL14 (methyltransferase 14), and WTAP (Wilms tumor-associated protein) writer complexes in the nucleus, and regulates the efficiency of mRNA splicing, processing, and metabolism. Importantly, nuclear YT (structure formed by the YT521-B protein) bodies that contain the YTHDC1 reader protein were already discovered in the late 1990s.^98^ YTHDC1-deficient leukemia cells exhibited significantly delayed disease development in patient-derived xenotransplantation models of AML.^12^ Interestingly, Glu-rich N-terminal and Arg-Pro-rich C-terminal IDRs of YTHDC1 facilitated its nuclear compartmentalization in AML cells, but no such condensates were found in cord blood-derived CD34+ cells that generally express lower levels of YTHDC1. Strikingly, the formation of YT bodies was regulated by m^6^-A-methylated RNA that was bound via the YTH domain but also by the IDRs of YTHDC1. Finally, the authors demonstrated that YT bodies promote the expression of m^6^A-methylated mRNAs, among which the *MYC* mRNA was identified as a direct target of YTHDC1.

Another example that illustrates the role of RNA-protein condensates in AML pathology is provided by mutations of splicing factors, including SF3B1, U2AF1, and ZRSR2, which emerge as recurrent drivers of myeloid malignancies, in particular during the transition from myelodysplastic syndromes (MDS) to AML.^99^ Dissection of mRNA binding at single nucleotide resolution of mutated U2AF1 revealed de novo 3′ splice site contacts in transcripts coding for proteins with low-complexity domains that promote the formation of cytoplasmic stress granule condensates.^100^ Higher expression of stress granule components in U2AF1-mutated cells was confirmed by mass spectrometry and immunofluorescence staining.^101^ Upregulation of stress granule components upon U2AF1 mutation was further confirmed by single-cell RNA sequencing of patient-derived *U2AF1*-mutant MDS/AML blasts compared with wild-type cells from the same patient. Notably, *U2AF1*-mutant cells were more resistant to sodium arsenite-induced stress than their wild-type counterparts, suggesting that enhanced formation of stress granule condensates potentially increases stress adaptation and contributes to clonal advantage in MDS/AML.^101^

## AML-ASSOCIATED BIOMOLECULAR CONDENSATES: POTENTIAL THERAPEUTIC TARGETS?

**Figure 3.**
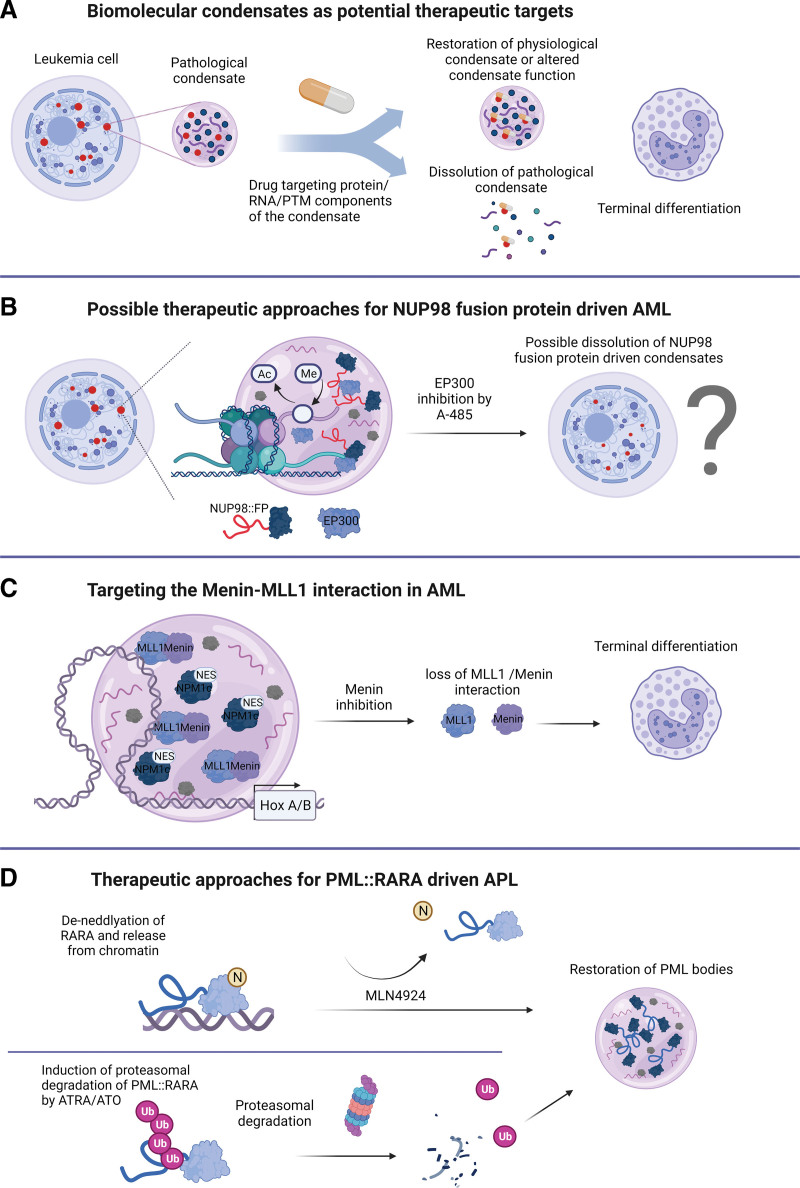
**Therapeutic options to target oncogenic condensates in AML.** (A) Leukemic cells harboring pathological condensates can be targeted by drugs targeting condensate protein/RNA components or their posttranscriptional/ translational modifications. Drug partitioning into condensates can induce the restoration of physiological condensates, alter condensate function or dissolve pathological condensates, and ultimately lead to terminal differentiation. (B) The histone deacetylase EP300 localizes to nuclear condensates and treatment with the EP300 inhibitor A-485 leads to dissolution of condensates into more numerous and smaller structures. As EP300 interacts with NUP98-fusion proteins that form pathological condensates, A-485 treatment might affect the formation of NUP98-fusion condensates with potential implications in therapy development. (C) Inhibition of Menin leads to loss of the Menin-MLL1 interaction, which is required for transcriptional activity of target HOX genes in many AML subtypes. (D) In PML::RARA-driven APL, de-neddylation of the RARA moiety by MLN4924 leads to the release of the fusion protein from chromatin and restoration of PML bodies. Induction of proteasomal degradation of PML::RARA by ATRA/ATO causes restoration of physiological PML bodies. AML = acute myeloid leukemia; ATO = arsenic trioxide; ATRA = all-trans retinoic acid; PML = promyelocytic leukemia protein.

The above-mentioned studies highlight that formation or disruption of biomolecular condensates plays a fundamental role in the biology of AML. As condensates drive a plethora of cellular logistics, interfering with the general physicochemical properties of biomolecular condensates might cause a nonspecific breakdown of cellular structures and would therefore not be feasible. The aliphatic alcohol 1,6-hexanediol is frequently used to disrupt condensates in cells in experimental settings. However, this compound is not suited as a therapeutic agent due to its broad effects. It severely impairs the activity of kinases, phosphatases, and DNA polymerases already at low concentrations.^102^ Therefore, interfering with the formation of oncogenic biomolecular condensates by specifically targeting their protein and RNA constituents could offer novel strategies for therapeutic intervention. Condensate formation is often regulated by PTMs that are catalyzed by condensate-resident enzymes for which small molecule inhibitors are readily available.^33,103,104^ Furthermore, RNA modifications and/or concentrations play key roles in the formation and dissolution of many subcellular compartments^14^; therefore, RNA-based therapeutics may also be able to target biomolecular condensates. These include antisense oligonucleotides (ASOs) that target mRNAs through complementary base pairing, or RNA aptamers, which bind their target based on their 3-dimensional structure.^105^ Moreover, the restoration of healthy condensates, which are disrupted in disease, may be equally beneficial (Figure 3A). These strategies for therapeutic interference with condensate function are mostly based on the targeting of proteins or nucleic acids, which are critical to form condensates that are associated with a particular pathology.

Alternatively, altering condensate properties and function can also be achieved through selective partitioning of drugs called phase modulators^106–108^ (Figure 3A). In a recent study, it was shown that the chemotherapeutic agent cisplatin is enriched in transcriptional condensates formed by the Mediator protein complex, thereby exhibiting a selective effect on oncogene enhancers that are bound by the mediator component MED1 (mediator complex subunit 1).^109^ Selective concentrating behavior of cisplatin was suggested to appear due to interactions with aromatic amino acids in MED1 protein. In the same study, the small molecule BET bromodomain inhibitor JQ1 (which selectively interferes with binding to acetylated lysine residues) partitioned not only into condensates formed by BRD4 (bromodomain-containing protein 4), but also into those formed by its nontarget proteins NPM1 and MED1, suggesting that condensate partitioning could be target-independent.^109^ In this case, binding of small molecules not only interferes with the function of BET proteins as an epigenetic reader but also with their role as a critical component for biomolecular condensate formation.^109,110^

Ma et al^111^ demonstrated that hydrophobic interactions between the IDRs of the transcriptional coactivator EP300 and the transactivation domain of NF-kappa-B facilitate their cocondensation. Furthermore, treatment with the inhibitor A-485 targeting the EP300’s enzymatic activity caused disassembly of EP300-containing condensates into smaller and more numerous compartments. As EP300 interacts with ≥400 TFs and has been detected at the promoters of >15,000 genes in human cells,^112,113^ the composition and function of most EP300 condensates remains unclear. For instance, AML-associated NUP98 fusion proteins were shown to recruit CREBBP/EP300 into condensates via interaction with FG repeat-containing IDRs to promote histone acetylation at promoters of protooncogenes.^61^ It would be, therefore, of interest to explore the effect of A-485 on condensates nucleated by different NUP98 fusion proteins. Given the antileukemic activity of another small molecule CREBBP/EP300 inhibitor (I-CBP112) in AML cells with NUP98 fusions, one may predict that this drug also impairs the formation and/or function of NUP98-fusion protein-containing condensates (Figure 3B).^114^ Importantly, as condensates can also be induced by functionally impaired NUP98-fusion proteins, CREBBP/EP300 inhibition likely impairs the oncogenic activity of NUP98-fusion through inhibitory effects on condensate constituents rather than on condensate formation per se.^62^

The interaction between Menin and MLL1 is critical for various AML subtypes due to the pivotal role of MLL1 in the regulation of HOX cluster genes and other factors that maintain the stemness of hematopoietic progenitor cells. Interference with the Menin-MLL1 interaction via small molecule inhibitors results in loss of chromatin occupancy of MLL1 and MLL-fusion proteins, causing differentiation and apoptosis of AML blasts (Figure 3C).^115,116^ Notably, NUP98 fusion proteins were shown to interact with MLL1, and MLL1 was critical for the maintenance of NUP98-rearranged AML.^69^ More recent work showed that MLL1-Menin inhibition evicts NUP98 fusion proteins from chromatin, thereby impairing leukemic transformation.^75^ Menin inhibitors are currently investigated in AML subtypes characterized by aberrant *HOXA*/*MEIS1* expression, including NPM1-mutated AML.^111^ Interestingly, Wang et al^89^ recently showed that nuclear NPM1c-containing condensates hijack MLL/Menin complexes to drive expression of HOXA and *MEIS1* genes. Upon combined treatment with MLL1-Menin inhibitors and XPO1 inhibitors such as Selinexor or Eltanexor, NPM1c nuclear condensates failed to maintain active transcription of *HOXA*/*MEIS1* genes. These data show that a thorough characterization of the composition of disease-associated biomolecular condensates can unravel potentially actionable interactions/targets.

As outlined earlier, PTMs can influence condensate properties. While it is known that AML is often driven by cooperating mutations that hyperactivate kinase signaling pathways, the effects of aberrantly activated signaling pathways on AML-associated biomolecular condensation have not been addressed. It was reported that oncogenic receptor tyrosine kinase fusions form cytoplasmic membrane-less protein granules in solid cancer cells, and these structures are essential for the activation of downstream signals.^117^ Importantly, biomolecular condensation was found to be dependent on the oligo/tetramerization motifs present in the fusion protein. Although this has not been investigated, oncogenic kinase signaling might operate through related mechanisms in hematological malignancies. The BCR::ABL fusion protein, which is the hallmark driver of chronic myeloid leukemia and some forms of acute lymphoblastic leukemia, represents the fusion of an oligomerization motif to a protein kinase domain. Notably, localization of the BCR::ABL fusion protein to stress granules was proposed to be essential for its oncogenic activity.^118^ Whether other signaling pathways that are hyperactivated through AML-associated mutations such as *FLT3-ITD* or *N/K-RAS* also involve alterations in biomolecular condensates remains to be tested. Oncogenic condensates that are induced by altered signaling pathways could represent potential biomarkers for successful therapeutic targeting of protein kinases by small molecule inhibitors. In addition, several protein kinases have direct roles in the regulation of gene expression. For instance, the cyclin-dependent kinase 9 (CDK9) is frequently dysregulated in AML and activates leukemic gene expression programs via the mTOR signaling pathway.^119^ CDK9 was previously shown to localize to biomolecular condensates,^120^ but it is unclear whether pharmacological inhibition of CDK9 and the related kinases CDK7, CDK12 nor CDK13 has antileukemic potential via disruption of specific biomolecular condensates.^121,122^

The restoration of tumor suppressive condensates is another strategy that is actively studied in the context of AML. Therapeutic targeting of APL by all-trans retinoic acid and/or arsenic trioxide (ATO) results in the proteasomal degradation of PML::RARA and restoration of tumor-suppressive PML bodies.^76^ Similarly, the neddylation inhibitor MLN4924 neutralized enhanced chromatin binding of PML::RARA, leading to the reformation of physiological PML bodies^80^ (Figure 3D). Furthermore, primary leukemia cells from AML patients expressing mutated NPM1c also exhibit aberrant PML body formation. NPM1c binds PML through the formation of disulfide bonds, resulting in the disassembly of PML bodies.^123^ Clinical observations indicated that inhibition of nucleolar transcription of ribosomal DNA by actinomycin D (Act-D) could induce complete remissions and even cure NPM1c^+^ AML patients.^124^ Molecular studies demonstrated that treatment with Act-D disrupted NPM1c-PML complexes, restored PML bodies, and prevented aberrant clonogenic activity of NPM1c^+^ AML cells. Moreover, the re-establishment of PML bodies induced senescence of leukemia cells but not of normal bone marrow progenitors in vivo.^123^ More recently, it has been shown that restoring PML body formation by ATO in JAK2^V617F^ myeloproliferative neoplasms could be beneficial in combination with standard therapies.^125^ Collectively, these findings suggest that restoring tumor suppressive biomolecular condensates, such as PML bodies could provide a powerful therapeutic strategy that is not restricted to APL but would likely be also effective in other myeloid malignancies (Figure 3D).

In summary, future in-depth analysis of biomolecular condensates induced by NPM1c, NUP98-, and/or KMT2A-fusion proteins and other AML oncoproteins may allow to dissect not only novel critical protein-protein interactions but also transcription-permissive gene loci that are prevalent in preleukemic clones and might be associated with a higher risk for progressing toward AML. In-depth dissection of the protein/RNA/DNA content and screens to identify chemical compounds that affect condensate formation and/or localization will help to define novel vulnerabilities as well as strategies for targeted intervention in AML and other cancers.

## LIMITATIONS AND CRITICISM OF THE BIOMOLECULAR CONDENSATION CONCEPT

Despite the growing interest in cancer-associated biomolecular condensates, it is important to mention that several researchers criticized the concept of biomolecular condensation to explain the formation of functional cellular compartments.^21,126^ Inspired by elegant studies on the nucleolus, many groups proposed condensation as a mechanism of compartmentalization across multiple cellular contexts. As a result, biomolecular condensate emerged as an umbrella term for membrane-less cellular compartments that are composed of proteins and nucleic acids, regardless of their size, function, mechanism of formation, and method of experimental study.^10^ The main criticism of this view is related to the assembly of biomolecular condensates, which was proposed to be driven by weak and unspecific interactions. Given that unspecific interactions of similar types cause strong competition for binding partners, it might be more favorable that site-specific interactions drive macromolecular concentration to establish and maintain the identity of cellular compartments.^126^ For instance, the kinetochore is a chromatin-associated multiprotein assembly that concentrates around 60 factors through site-specific interactions that depend on discrete binding interfaces, despite the presence of many IDR-carrying proteins.^127^ Site-specific interactions are extremely versatile,^128^ and their kinetics can often be misinterpreted and confused with unspecific interactions.^126^ Furthermore, artifacts associated with protein overexpression in many experimental models of AML^58^ and cancer in general could lead to a discrepancy between phenomena observed in experimental models versus physiological conditions. The currently used methods to study biomolecular condensates (Suppl. Table S2) are mostly unable to overcome these limitations. Future studies need to focus on endogenously expressed proteins in their native state or fused to genetically encoded tags that make them amenable to image-based and/or biochemical characterization.^129^

Another general limitation to study the role of AML-associated biomolecular condensates is that it is often difficult to provide direct proof for condensate-specific functions of candidate factors.^126^ When biomolecular condensates are perturbed through genetic or pharmacological inactivation of their components, it is challenging to discriminate whether the perturbation interferes with the cause (ie, the physicochemical properties of condensate components) or the consequence (ie, the function of the biomolecular condensate) as both are functionally connected. These challenges arise not only from limitations in experimental methods but also often from the lack of interdisciplinary approaches that this field requires to obtain a comprehensive understanding of condensate structure and function in disease.^130^ The need for better-defined experimental strategies has been recognized by Gao et al,^131^ who proposed to introduce comprehensive guidelines for the study of condensates that consist of their physical characterization and their functional analysis.

In conclusion, the field will need to increase its reliance on better cellular models and experimental methods to investigate the potential of perturbing biomolecular condensates as a valid therapeutic approach.

## AUTHOR CONTRIBUTIONS

ZJ & MA wrote, illustrated and edited; FG & JS developed the concept, wrote and edited the manuscript.

## DISCLOSURES

JS is an editor for HemaSphere. The authors have no conflicts of interest to disclose.

## SOURCES OF FUNDING

This work was supported by Swiss Cancer Research (KFS-5267-02-2021) to JS. Research in the Grebien laboratory is supported by the Austrian Science Fund (projects P-35628 and P-35298). All figures were created with BioRender.com.

## Supplementary Material


