# Supplementary material for: Biomolecular Condensates in Myeloid Leukemia: What Do They Tell Us?
Source: Hemasphere. 2023 Jun 27;7(7):e923. doi: 10.1097/HS9.0000000000000923 (PMC10306439; doi:10.1097/HS9.0000000000000923)
Supplement: Supplementary file 1 [file hs9-7-e923-s001.pdf]

Supplementary Table 1: Reported AML-associated biomolecular condensates

Disease	Gene products	IDRs	Visualization	Condensate components	Methodology	Cell (lines)	Overexpression/endogenous	Mouse models	Refs.
AML, MDS	NUP98-r	FG repeat		157 common interactors		No human AML cell line			[57]
	NUP98-NSD1		confocal microsc.	EP300, XPO1, SMARCA5 *et al.*	Proteomics	Mouse primary HSPC, 293T	Retroviral overexpression	BM reconstitution	[60] [62]
	NUP98-HOXA9		confocal microsc.	KMT2A, WDR5, MOF *et al.*	Proteomics	Mouse primary HSPC, 293T	Retroviral overexpression	BM reconstitution	[58]
AML, tAML	KMT2A-r	AHC domain	confocal microsc.	MEN1, DOT1L, LEDGF, PAF1C, *et al*.	Proteomics	THP1, NOMO1, MOLM13, MV4-11	Retroviral overexpression & endogenous	Transgenic & BM reconstitution	[71] [72] [73]
APL	PML-RARA	*RBCC	confocal microsc.	UBC9, SUMO1/2/3…	Immunoprecipitation	NB4	Endogenous proteins	Transgenic & BM reconstitution	[78] [80]
AML	NPM1c	A2-3, B1-2	confocal microsc.	KMT2A, ENL, AF4, AF9 et al.	LacO-array assay	OCI-AML3	Endogenous proteins	BM reconstitution & PDX*	[84] [89] [90]
AML, tAML	MN1	Poly-Glu strain	confocal microsc.	SMARCA4, SMARCC1, ARID1A…	Proteomics	UCSD-AML1, MUTZ-3, AMU-AML1	Endogenous proteins	BM reconstitution	[11]
AML	YTHDC1	Glu-rich (N-term.), Arg/Pro-rich (C-term.)	confocal microsc.	m6A modified RNA, YTHDC1...	*In vitro* reconstitution	THP1, NOMO1, MOLM13, OCI-AML3, KASUMI1	Endogenous proteins	BM reconstitution & PDX*	[12] [98]

* The RBCC domains (RING finger domain (R) followed by two cysteine-histidine-rich B-box domains (B) and an alpha-helical coiled-coil domain (CC)) XXX
* Patient-derived xenograft
