# Supplementary material for: Biomolecular Condensates in Myeloid Leukemia: What Do They Tell Us?
Source: Hemasphere. 2023 Jun 27;7(7):e923. doi: 10.1097/HS9.0000000000000923 (PMC10306439; doi:10.1097/HS9.0000000000000923)
Supplement: Supplementary file 2 [file hs9-7-e923-s002.pdf]

Jevtic, Allram et al., Supplementary Table 2

Methods	Description	References: Digital Object Identifier (DOI):
**Fluorescence microscopy**	Tracking *in vitro* reconstituted condensates (droplets) over time and testing their formation, dissolution kinetics, mechanistic properties in various physico-chemical conditions. *In vivo* condensates can be analyzed using single-molecule assays to determine the stoichiometry of condensate constituents.	https://doi.org/10.1016/j.tibs.2020.05.011
**Biochemical assays**	Identification of protein and RNA constituents within biomolecular condensates by using combined proteomic profiling and DNA/RNA-seq. Crosslinking assays can be used to identify protein-protein and protein-RNA interactions.	https://doi.org/10.1016/j.jprot.2012.04.030
**Optogenetic approaches**	Analyzing the biophysical properties of biomolecular condensates by fusing the photolyase homology region (PHR) domain of the *Arabidopsis thaliana* cryptochrome 2 (CRY2) to various IDR domains, enabling inducible and reversible condensation in living cells.	https://doi.org/10.1002/wsbm.1415
**NMR spectroscopy**	Acquiring atomic-resolution view on intra/interprotein interactions over various timescales providing the information on domain/loop motions of *in vitro* reconstituted condensates.	Https://doi.org/10.1042/EBC20220056
**Microfluidics**	Generating and high-throughput screening of thousands of well-defined microcompartments within a short time. Condensate composition can be manipulated by introducing additional molecules.	https://doi.org/10.1016/j.bbamcr.2020.118823
**Cryo-electron tomography**	Visualizing the condensate netowrk in 3D with nanometer resolution through repeating, aligning, and averaging acquired structures.	https://doi.org/10.1016/j.devcel.2020.09.003
